# P-1186. Significant Reduction in Disease Burden and a Shift in Clinical Diagnoses in Children Hospitalized with Respiratory Syncytial Virus (RSV) after Nirsevimab Implementation in Catalonia (Spain)

**DOI:** 10.1093/ofid/ofae631.1370

**Published:** 2025-01-29

**Authors:** Anna Creus-Costa, Maria Piñana, Aida Perramon-Malavez, Cristina Andrés, Victòria Rello-Saltor, Romy Rossich-Verdés, Ariadna Carsi-Durall, Sonia Cañadas-Palazón, Marc Tobeña-Rué, Sebastià González-Peris, Mónica Sancosmed-Ron, Rocío Rodrigo-García, Olalla Rodríguez-Losada, Esther Lera-Carballo, Núria Wörner-Tomasa, Alejandro Casquero-Cossío, Anna Vidal-Moreso, Zulema Lobato, Berta Pujol-Soler, Iris González, Núria López, Montserrat Ruiz-García, Sergio Flores, Neus Rius, Núria Visa-Reñé, Laura Geronès, Asuncion Mejias, Joan Balcells, Pere Soler-Palacín, Clara Prats, Jorgina Vila, Andrés Antón, Antoni Soriano-Arandes

**Affiliations:** Vall d'Hebron Barcelona Hospital Campus, Barcelona, Catalonia, Spain; Vall d'Hebron Barcelona Hospital Campus, Barcelona, Catalonia, Spain; Universitat Politècnica de Catalunya, Barcelona, Catalonia, Spain; Vall d'Hebron Barcelona Hospital Campus, Barcelona, Catalonia, Spain; Vall d'Hebron Barcelona Hospital Campus, Barcelona, Catalonia, Spain; Vall d'Hebron Barcelona Hospital Campus, Barcelona, Catalonia, Spain; Vall d'Hebron Barcelona Hospital Campus, Barcelona, Catalonia, Spain; Vall d'Hebron Barcelona Hospital Campus, Barcelona, Catalonia, Spain; Vall d'Hebron Barcelona Hospital Campus, Barcelona, Catalonia, Spain; Vall d'Hebron Barcelona Hospital Campus, Barcelona, Catalonia, Spain; Vall d'Hebron Barcelona Hospital Campus, Barcelona, Catalonia, Spain; Vall d'Hebron Barcelona Hospital Campus, Barcelona, Catalonia, Spain; Vall d'Hebron Barcelona Hospital Campus, Barcelona, Catalonia, Spain; Vall d'Hebron Barcelona Hospital Campus, Barcelona, Catalonia, Spain; Vall d'Hebron Barcelona Hospital Campus, Barcelona, Catalonia, Spain; Vall d'Hebron Barcelona Hospital Campus, Barcelona, Catalonia, Spain; Consorci Sanitari del Maresme, Hospital de Mataró, Mataró, Catalonia, Spain; Hospital Althaia Manresa, Manresa, Catalonia, Spain; Hospital General de Granollers, Granollers, Catalonia, Spain; Hospital de Terassa, Consorci Sanitari de Terrassa, Terrassa, Catalonia, Spain; Hospital Universitari del Mar, Barcelona, Catalonia, Spain; Hospital Universitari de Vic, Vic, Catalonia, Spain; Hospital Universitari Mútua de Terrassa, Terrassa, Catalonia, Spain; Hospital Universitari Sant Joan de Reus, Reus, Catalonia, Spain; Hospital Universitari Arnau de Vilanova, Lleida, Catalonia, Spain; Hospital de Palamós, Palamós, Catalonia, Spain; St Jude Children's Research Hospital, Memphis, TN; Vall d'Hebron Barcelona Hospital Campus, Barcelona, Catalonia, Spain; Vall d'Hebron Barcelona Hospital Campus, Barcelona, Catalonia, Spain; Universitat Politècnica de Catalunya, Barcelona, Catalonia, Spain; Vall d'Hebron Barcelona Hospital Campus, Barcelona, Catalonia, Spain; Hospital Universitario Vall d’Hebrón, Barcelona, Catalonia, Spain; Vall d'Hebron Barcelona Hospital Campus, Barcelona, Catalonia, Spain

## Abstract

**Background:**

RSV causes a significant burden including hospitalization in infants. Nirsevimab prophylaxis was implemented in October 2023 in Catalonia (Spain) for all children < 6-months-old to reduce RSV-associated morbidity. We aimed to define the impact of nirsevimab administration on clinical outcomes of hospitalized children with RSV infection.

**Figure:**
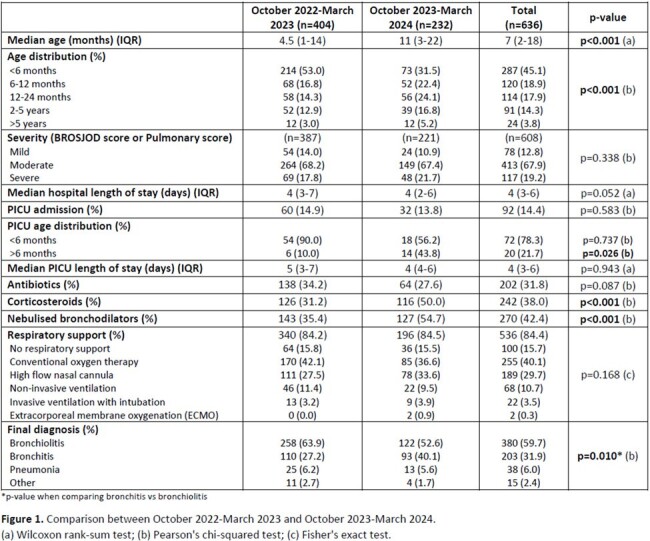

**Methods:**

We compared clinical data from RSV-associated hospitalizations in children < 16-years-old between October 2022 - March 2023 and October 2023 - March 2024. Respiratory samples were collected from 11 hospitals of the COPEDICAT network (https://www.copedicat.cat). Laboratory confirmation was performed by a real-time multiplex RT-PCR-based assay (Allplex Respiratory Panel Assay, Seegene, South Korea) or antigen test. Disease severity was defined by the pulmonary score and/or need for PICU care.

**Results:**

We included 636 hospitalized patients (63.5% in 2022-23 and 36.5% in 2023-24), mostly with lower respiratory tract infections (97.6%). Median age in the 2022-23 season was 4.5 vs 11 months in the following season (p< 0.001). The proportion of children < 6-months-old was also significantly lower in 2023-24 (53.0% vs 31.5%, p< 0.001) (**Figure 1**). There was a 42.6% reduction in hospitalizations in 2023-24 compared with the previous season; most of the hospitalized children in the 2023-24 season had not been immunized with nirsevimab (72.5%). In 2023-24, there was a higher proportion of bronchitis (40.1% vs 27.2%, p< 0.001) and children treated with bronchodilators and corticosteroids (both p< 0.001) compared to the previous season. The number of PICU admissions decreased by half (60 vs 32), but its percentage in relation to total hospital admissions did not change significantly (14.9% vs 13.8%, p=0.58). No significant differences were found in the clinical score at admission or respiratory support.

**Conclusion:**

After the introduction of nirsevimab in infants < 6-months-old, children admitted to catalan hospitals were older and the proportion of bronchitis was significantly higher than in the previous season. In addition, there was a reduction of almost 50% RSV-associated hospitalizations and PICU admissions compared to the previous season; most of the hospitalized children had not been immunized with nirsevimab.

**Disclosures:**

**Asuncion Mejias, MD, PhD, MsCS**, Astra-Zeneca: Advisor/Consultant|Astra-Zeneca: Honoraria|Enanta: Advisor/Consultant|Janssen: Advisor/Consultant|Janssen: Grant/Research Support|Merck: Advisor/Consultant|Merck: Grant/Research Support|Moderna: Advisor/Consultant|Pfizer: Advisor/Consultant|Pfizer: Honoraria|Sanofi-Pasteur: Advisor/Consultant|Sanofi-Pasteur: Honoraria

